# Daphnetin modulates GLP-1R to alleviate cognitive dysfunction in diabetes: implications for inflammation and oxidative stress

**DOI:** 10.3389/fphar.2024.1438926

**Published:** 2024-08-27

**Authors:** Feng Liang, Xinyi Tian, Lining Ding

**Affiliations:** ^1^ Department of Clinical Laboratory, Key Laboratory of Clinical Laboratory Diagnosis and Translational Research of Zhejiang Province Wenzhou, The First Affiliated Hospital of Wenzhou Medical University, Zhejiang, China; ^2^ College of Chemistry and Pharmacy, Northwest A&F University, Xianyang, China; ^3^ Guangdong Pharmaceutical University, Guangzhou, China

**Keywords:** daphnetin, diabetes cognitive dysfunction, pharmacological effects, GLP-1 receptor, inflammation and oxidative stress

## Abstract

Daphnetin exerts certain pharmacological function on a variety of diseases, but its role in diabetic cognitive dysfunction has not been elucidated. In this study, we carried a series of pharmacological studies of GLP-1R with daphnetin. In rats and PC12 cells, we found that daphnetin could alleviate diabetic cognitive dysfunction and increase the expression level of GLP-1R. Additionally, the anti-diabetic cognitive dysfunction effect of DAP was accompanied by the inhibition of inflammation and oxidative stress. Further in-depth studies demonstrated that the inhibition GLP-1R enhanced the protective effect of daphnetin, whilst, the overexpression of GLP-1R weakened the protective effect of daphnetin. These results indicated that daphnetin protects diabetes cognitive dysfunction by regulating GLP-1R-mediated inflammation and oxidative stress, act as a GLP-1R agonist. The study further demonstrated that daphnetin has great value in preventing cognitive dysfunction in type 2 diabetes, and GLP-1R is a key potential target for the treatment of related diseases.

## 1 Introduction

Diabetes is a metabolic disease that poses a serious threat to human health (Zheng et al., 2018). It is estimated that by 2045, 629 million people worldwide will be affected by diabetes (Federation, 2021). Diabetes cognitive dysfunction is a complication of diabetes that causes patients to have impaired learning, mental flexibility, and executive function, which seriously affects their quality of life (Munshi, 2017). In Additionally, diabetes cognitive impairment is associated with an increased risk of mental illnesses such as dementia, Alzheimer’s disease (AD), and depression (Kim, 2019; Srikanth et al., 2020). However, there is currently no specific drug that effectively treats or intervenes in diabetic cognitive impairment. It is imperative to identify and treat diabetic cognitive impairment and develop effective targeted drugs.

At present, the molecular mechanism of diabetic cognitive dysfunction has not been elucidated, and it may involve multiple factors, such as oxidative stress, neuroinflammation, endothelial cell dysfunction, etc (Bhatia and Singh, 2021; Chen et al., 2024). Recently, the inflammation and oxidative stress theory of diabetic cognitive dysfunction has been increasingly recognized by researchers, and inflammation and oxidative stress may be involved in its pathological progression. For example, Tian et al. (2024) found that the levels of oxidative stress-related factors (NOX4, iNOS superoxide anion, peroxides and hydrogen peroxide), and inflammation mediators (IL-6, IL-1β, NLRP3, ASC and caspase1) were higher in diabetes cognitive dysfunction mice. Thus, anti-inflammatory and anti-oxidative stress may be an effective way to improve diabetic cognitive dysfunction.

The utilization of plant-derived phytochemicals for disease intervention has increasingly become a focal point in contemporary research. For example, Kaempferol has been identified as an effective agent in mitigating diabetic inflammation through its suppression of the RhoA/Rho-kinase signaling pathway (Sharma et al., 2019). Furthermore, Kaempferol exhibits promising potential in alleviating renal damage associated with diabetes by reducing fibrosis (Sharma et al., 2020). Daphnetin is a natural coumarin derivative with multiple pharmacological effects, including antioxidant, anti-inflammation, anti-apoptosis (Guo et al., 2023; Yang et al., 2022). Currently, the potential therapeutic effects of daphnetin are becoming increasingly clear. Daphnetin prevented silica-caused damage via inhibiting PI3K/Akt-mediated inflammation and fibrosis (Yang et al., 2024). Additionally, daphnetin has been reported is useful for treating diabetes. Xu et al. (2019) stated that daphnetin alleviated high-glucose-caused oxidative stress and inflammation in human glomerular mesangial cells (Xu et al., 2019). Daphnetin could against STZ-caused β-cells damage through inhibiting apoptosis (Vinayagam and Xu, 2017). However, there are few studies on the effect of daphnetin on alleviating diabetes cognitive dysfunction, which deserves further investigation.

Glucagon-like peptide-1 receptor (GLP-1R) is an incretin hormone that has been shown to improve memory deficits in a variety of diseases (Liarakos et al., 2023; Sharma et al., 2018), including bipolar disorder, diabetes-related depression and cognitive impairment (Cooper et al., 2023), and depression (Dumiaty et al., 2024). Additionally, GLP-1R also plays a critical role in regulating inflammation and oxidative stress. For example, Morrow have reported that the agonist of GLP-1R could activate inflammation and promote the production of inflammatory factors (Morrow et al., 2024). Knockout of GLP-1R could block the activation of Nrf2 and inhibit inflammation and oxidative stress (Tu et al., 2023).

To date, the molecular mechanism of daphnetin in diabetic cognitive dysfunction is unclear. In this study, we explored the protective effect of daphnetin on diabetic cognitive dysfunction and the potential regulatory mechanism between GLP-1R-mediated oxidative stress and inflammation.

## 2 Methods and materials

### 2.1 Chemicals

Daphnetin (HY-N0281) and streptozotocin (NSC-85998) were obtained from MedChemexpress (New Jersey, United States). For cell experiments, daphnetin were dissolved in DMSO (<0.1%) and 0.5% sodium carboxymethylcellulose (CMC-Na) for the *in vitro* and *in vivo* experiments, respectively. Methylglyoxal solution (M0252) was purchased by Sigma-Aldrich.

### 2.2 Cell culture

PC12 cells (CRL-1721) was purchased from American type culture collection. PC12 cells are seeded in 75 cm^2^ cell culture flasks and cultured in an incubator at 37°C in an incubator at 5% CO2 and subculture at a ratio of 1:4 when the cell density reaches 10^6^.

### 2.3 Cell treatment

PC12 cells were seeded in 96-well plates or 6-well plates. After 24 h of growth, basic medium containing different concentrations of daphnetin was added. After 12 h of incubation, the medium was replaced with basic medium containing 2 mM methylglyoxal. After 12 h of incubation, measured the cell viability and extracted total RNA.

### 2.4 Transfection of plasmids and siRNA

GLP-1R overexpression plasmids (pc-GLP-1R) and pcDNA3.1 were obtained from GeneCopoeia (Rockville, MD, United States). Small interfering RNA (si-GLP-1R) and siRNA-NC were purchased by RiboBio Co., Ltd. (Guangdong, China). The reagent used for transfection was Lipofectamine 3,000 (Thermo Fisher Scientific).

### 2.5 Animal

Fifty Sprague Dawley rats (male, 180–200 g, 2 months) were purchased from Taconic Biosciences and housed in a standard animal room. After a 2 weeks acclimation period, they were randomly divided into five groups. We constructed a diabetic rat model using a previously reported method, administering a dose of 30 mg/kg intraperitoneally to induce type 1 diabetes (Furman, 2021). Group 1: Control group, orally administered 0.5% CMC-Na solution; Group 2: Diabetes model group; Group 3: Diabetes model +10 mg/kg daphnetin (orally administered); Group 4: Diabetes model +20 mg/kg daphnetin (orally administered); Group 5: Diabetes model +30 mg/kg daphnetin (orally administered). The dose of daphnetin was selected based on previous studies (Hang et al., 2022; Li et al., 2020; Liu et al., 2016). After 12 weeks, the rats were deeply anesthetized according to the previous procedure (Hao et al., 2019), and the blood, brain, cortex, and hippocampus tissues were collected, and the other related indicators such as body weight were measured and recorded. All procedures were performed by the National Institutes of Health Guide for Care and Use of Laboratory Animals and Northwest A&F University (N81803231).

### 2.6 Immunohistochemistry

Immunohistochemistry was used to detect the expression of GLP-1R in brain tissues of rats in different groups (Qi et al., 2019). In brief, brain tissue sections were dewaxed and incubated with rabbit anti-GLP-1R overnight. The next day, incubated the sections with TRITC-conjugated goat anti-mouse IgG for 1 h, followed by counterstaining with DAPI and observed under a fluorescence microscope and photographed.

### 2.7 qRT-PCR

RT-PCR experiments were performed according to previously reported methods (Wang et al., 2017). Briefly, total RNA was extracted from PC12 cells or brain tissues, reverse transcribed into cDNA, and then the mRNA quantification experiment was performed using commercial kits (TaKaRa, Wuhan, China).

### 2.8 ELISA kits

The levels of antioxidant enzyme activities and inflammatory factors were measured by commercial kits (Algefare et al., 2024). SOD, GPx, GSH, GSSH, CAT, TAC kits were all obtained from Navand Salamat Company (Urmia, Iran). MDA kits was purchased from Teb Pazhouhan Razi Company (Tehran, Iran). TNF-α, IL-1β, IL-6 kits were obtained by Krishgen Biosystem Company (Shanghai, China).

### 2.9 Blood glucose determination

The method for measuring blood glucose was referred to the previous literature (Xu et al., 2020). After administration, the rats were fasted for 6 h, and blood glucose levels were measured by blood samples collected from the tail vein.

### 2.10 Behavioral testing

We followed the methods reported by previous researchers and used the elevated plus maze test, open field test and novel object recognition to evaluate the learning and cognitive abilities of rats (Lin et al., 2024; Walf et al., 2009). In brief, the open field test is to place the rat in the center of a box, use a camera to record the rat’s activity trajectory within 5 min, and record the distance in the central area and the time in the central area. For elevated plus maze places the rat in the central area and records the rat’s activity trajectory within 5 min. Lastly, analyzed the results by SuperMaze software. For novel object recognition, rats were putted in a box to adapt for 10 min, then put two identical cylinders in the box for 1 h of training, then take one of the cylinders out and replace it with a new cube, record the time of rats spend exploring the new and old objects within 10 min, and calculate the index. Recognition Index = Time of exploring new objects/Time of exploring new objects and old objects. For Y maze test, rats were placed in the center of the maze and allowed to explore freely for 5 min. The number of times the rats entered each arm and the number of spontaneous alternations (continuous entry into three different arms) were recorded. The alternation index = number of spontaneous alternations/(number of arm entries-2) (Alotayk et al., 2023).

### 2.11 Date analysis

All data were expressed as mean ± SD and statistically analyzed using GraphPad Prism V6.0. Differences between groups were analyzed by one-way analysis of variance (*p* < 0.05 were considered significant).

## 3 Results

### 3.1 Daphnetin prevented diabetes cognitive dysfunction in rats

**FIGURE 1:**
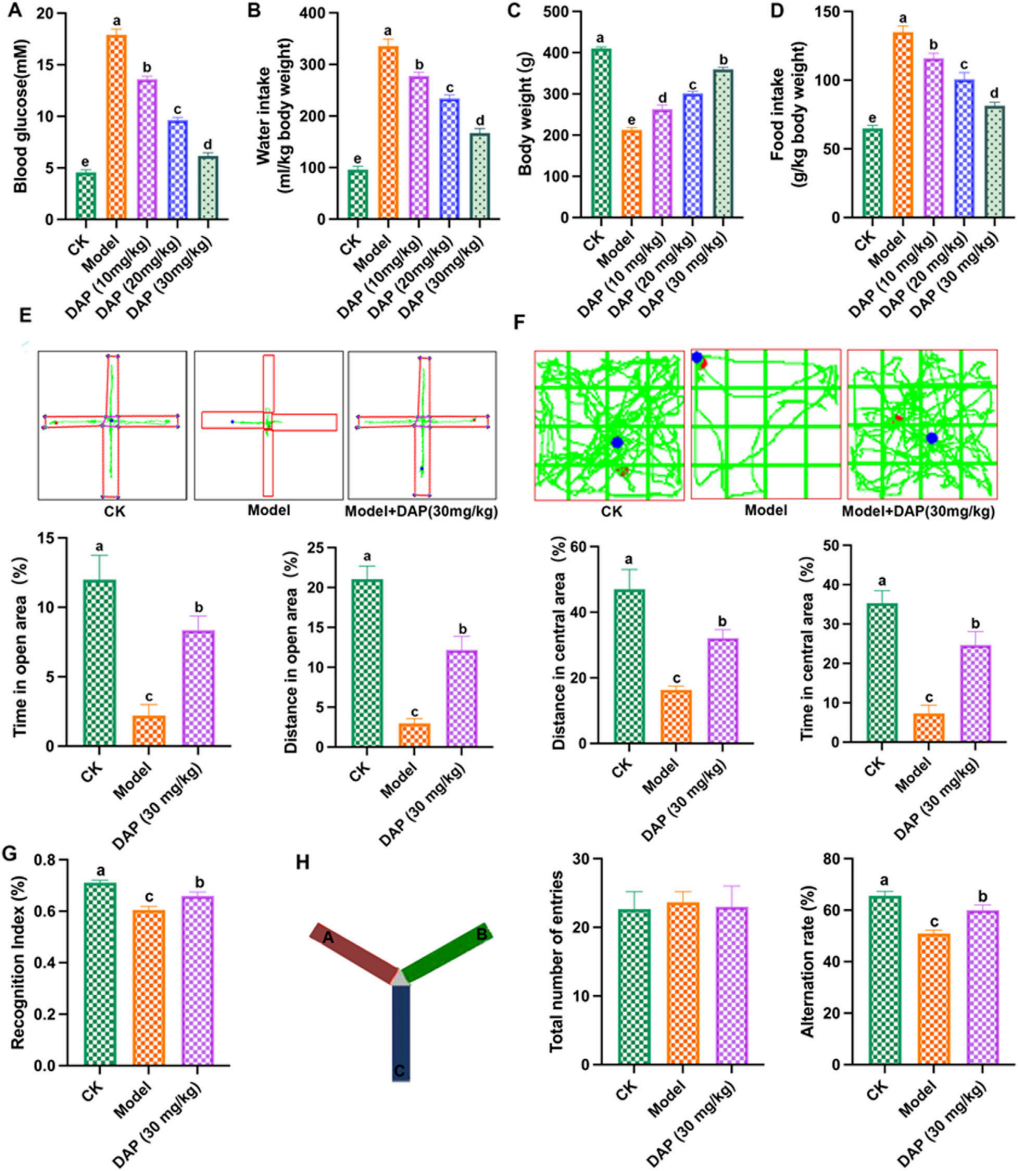
Daphnetin prevented diabetes cognitive dysfunction in rats. **(A)** blood glucose; **(B)** water intake; **(C)** body weight; **(D)** food intake; **(E)** elevated plus maze; **(F)** Open field test; **(G)** Novel object recognition test; **(H)** Y maze test. Different letters **(A–E)** denote a significant difference between groups (n = 10) (*p* < 0.05).

In order to investigate the role of daphnetin on diabetes cognitive dysfunction, we established the STZ-induced diabetes rat model and daphnetin treatment rat model. As shown in Figures 1A–D, STZ-treated increased the blood glucose, water intake and food intake, and decreased the body weight of rats, compared to the rats of control group, while daphnetin at doses of 10, 20 and 30 mg/kg decreased the blood glucose and the intakes of water and food, and increased the body weight of diabetes cognitive dysfunction rats. Furthermore, we found that the protective effect of daphnetin becomes more significant as the dose increases, and 30 mg/kg has the best protective effect. Thus, we subsequently selected daphnetin (30 mg/kg) for in-depth research. In addition, behavioral experiments were conducted to evaluate the effect of daphnetin on diabetes cognitive impairment. Elevated plus maze results showed that daphnetin increased the time of open area and distance of open area of diabetes cognitive dysfunction rats (Figure 1E). Open field test results showed that daphnetin upregulated the distance in central area and time in central area of diabetes cognitive dysfunction rats (Figure 1F). Novel object recognition test (Figure 1G) and Y maze test (Figure 1H) showed that daphnetin increased the recognition index and decreased alternation rate of diabetes rats. These results indicated that daphnetin could attenuate diabetes cognitive dysfunction in rats.

### 3.2 Daphnetin attenuated oxidative stress of diabetes cognitive dysfunction rats

**FIGURE 2:**
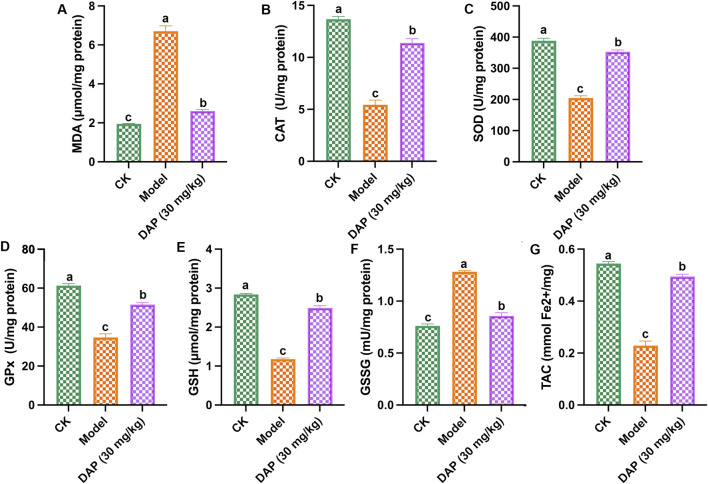
Daphnetin attenuated oxidative stress of diabetes cognitive dysfunction rats. **(A)** Malondialdehyde (MDA) content; **(B)** Catalase (CAT) activity; **(C)** Superoxide dismutase (SOD) activity; **(D)** Glutathione peroxidase (GPx) activity; **(E)** Glutathione (GSH) activity; **(F)** Glutathione disulfide (GSSH) activity; **(G)** Total antioxidant capacity (TAC) content. Different letters **(A–C)** denote a significant difference between groups (n = 6) (*p* < 0.05).

As shown in Figure 2, STZ-treated caused oxidative stress in rat brain, which increased the MDA content and GSSH content, and reduced the levels of SOD, CAT, GSH, Gpx and TAC, while daphnetin-treated inhibited these changes in oxidative stress-related medicators, which indicated that daphnetin could mitigate oxidative stress of diabetes cognitive dysfunction rats.

### 3.3 Daphnetin alleviated inflammation of diabetes cognitive dysfunction rats

**FIGURE 3:**
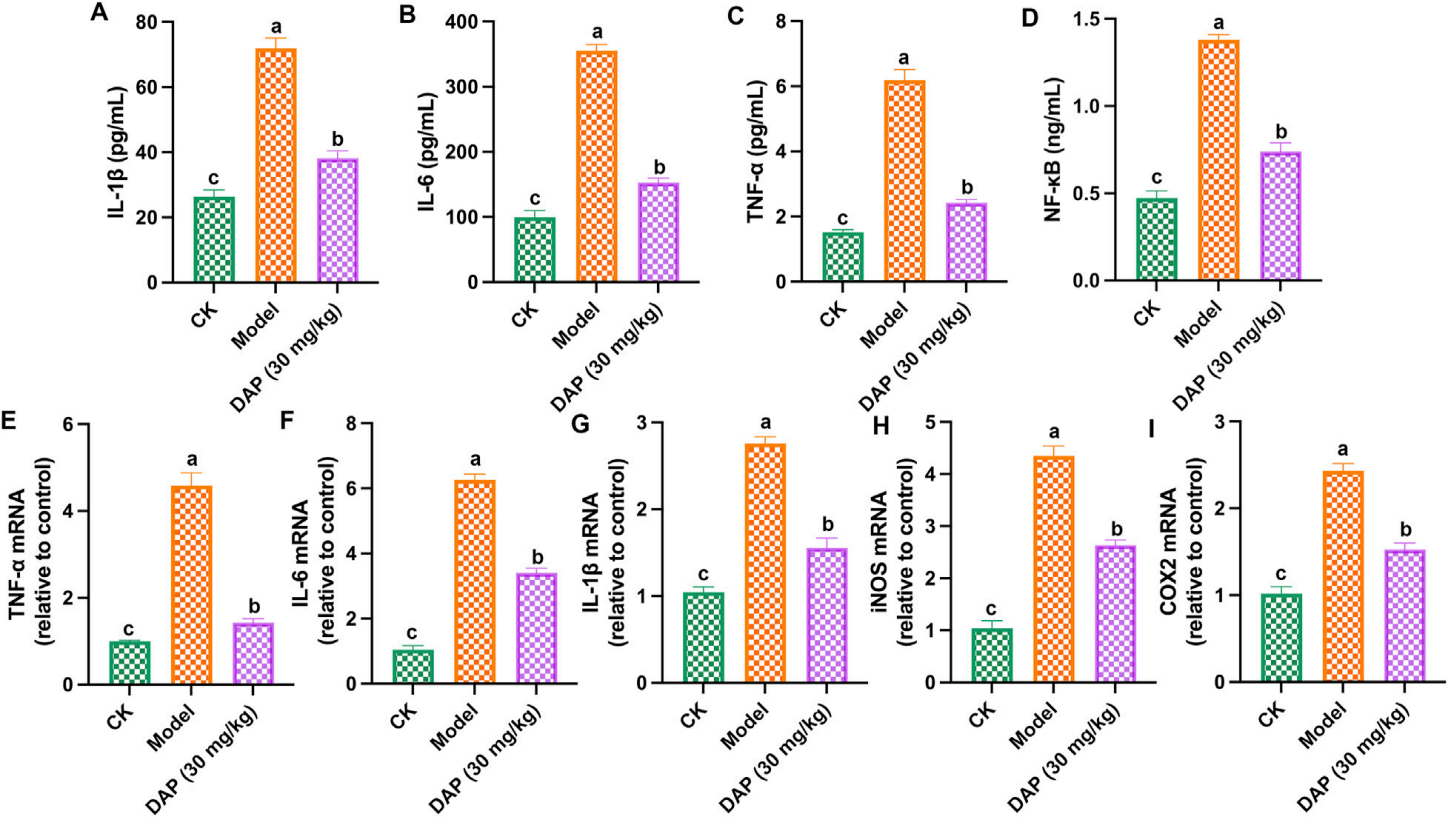
Daphnetin alleviated inflammation of diabetes cognitive dysfunction rats. **(A)** Interleukin-1 beta (IL-1β) content; **(B)** Interleukin-6 (IL-6) content; **(C)** Tumor necrosis factor Alpha (TNF-α) content; **(D)** NF-κB content; **(E)** TNF-α mRNA; **(F)** IL-6 mRNA; **(G)** IL-1β mRNA; **(H)** Inducible nitric oxide synthase (iNOS) mRNA; **(I)** Cyclooxygenase 2 (COX2) mRNA. Different letters **(A–C)** denote a significant difference between groups (n = 6) (*p* < 0.05).

Figure 3 showed that daphnetin could alleviate STZ-caused inflammation in rat brain, which reduced the contents of IL-1β, TNFα, IL-6 and NF-κB, and the mRNA expressions of IL-1β, TNFα, IL-6, iNOS and COX2. These results demonstrated that daphnetin alleviated inflammation of diabetes cognitive dysfunction rats.

### 3.4 GLP-1R is a key factor in daphnetin’s anti-diabetic cognitive dysfunction

**FIGURE 4:**
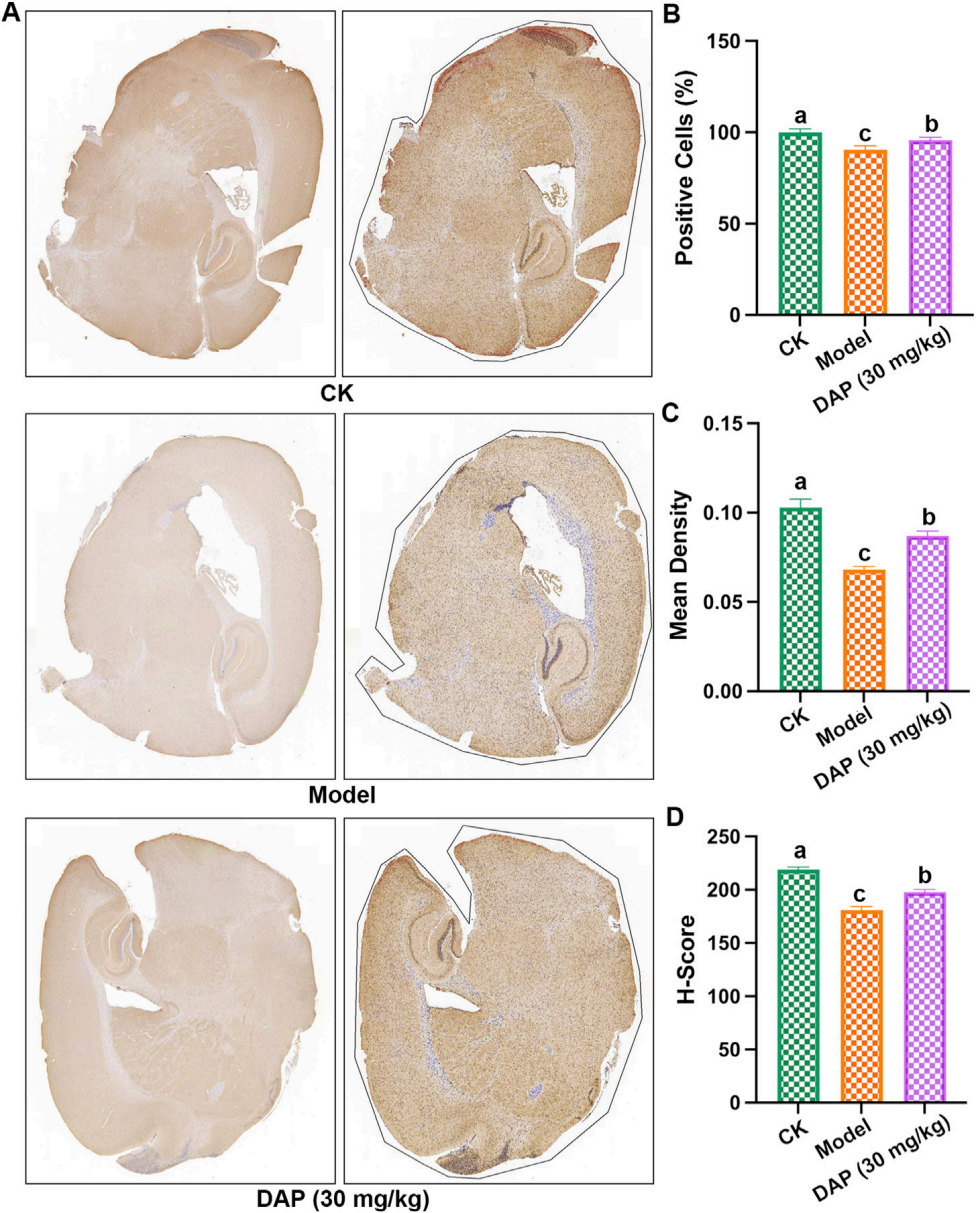
GLP-1R is a key factor in daphnetin’s anti-diabetic cognitive dysfunction. **(A)** Immunohistochemistry image of the whole brain; **(B)** positive cells in the whole brain; **(C)** Mean density; **(D)** Histochemistry score (H-score). Different letters **(A–C)** denote a significant difference between groups (n = 3) (*p* < 0.05).

The results of immunohistochemistry experiments (Figure 4) showed that STZ-treated decreased the positive cells, mean density and H-score of GLP-1R, and daphnetin-treated increased these levels, which indicated that daphnetin-treated increased GLP-1R level of diabetes cognitive dysfunction rats.

### 3.5 Inhibition of GLP-1R weakened the protective effect of daphnetin

**FIGURE 5:**
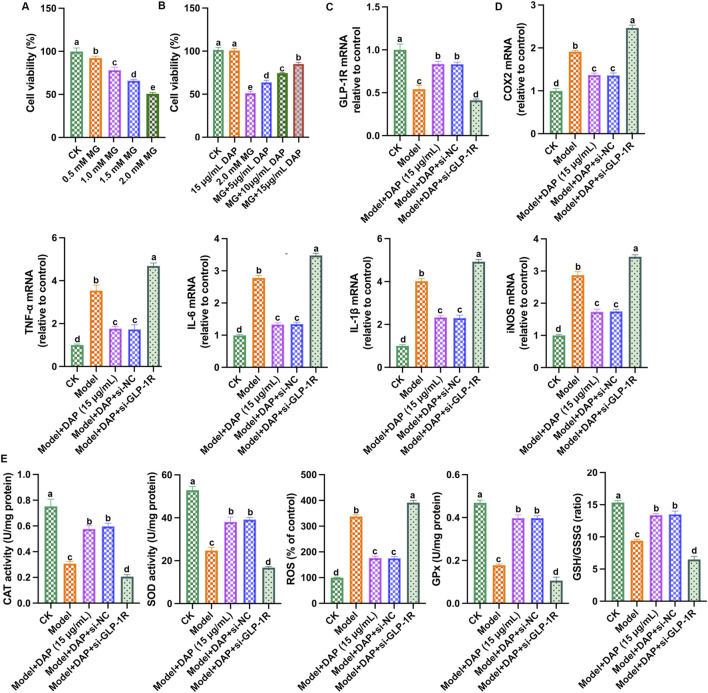
Inhibition of GLP-1R weakened the protective effect of daphnetin. **(A)** cell viability; **(B)** cell viability; **(C)** GLP-1R mRNA expression; **(D)** mRNA expressions of COX2, TNFα, IL-6, IL-1β and iNOS; **(E)** Levels of SOD activity, CAT activity, ROS content, GPx content and GSH/GSSG ratio. Different letters **(A–D)** denote a significant difference between groups (n = 6) (*p* < 0.05).

According to previous study, we constructed PC12 cell models using methylglyoxal to explore the protective effect of daphnetin. As shown in Figure 5A, as the concentration of methylglyoxal increased, PC12 cell viability gradually decreased, and the cell viability dropped to approximately 50% at the methylglyoxal dose of 2.0 mM. As shown in Figure 5B, compared with the model group, the cell viability of PC12 cells increased accordingly as the daphnetin concentration increased, and the daphnetin concentration of 15 μg/mL had the best protective effect. These results demonstrated that diosgenin attenuates diabetic cognitive dysfunction in methylglyoxal-treated PC12 cells in a dose-dependent manner. Subsequently, 2.0 mM of methylglyoxal and 15 μg/mL of daphnetin were selected as the subsequent experimental concentrations.

So far, we have proven that daphnetin mitigated diabetes cognitive dysfunction, inhibited oxidative stress and inflammation, and GLP-1R is a key regulatory factor. Therefore, we aimed to demonstrate whether the protective effect of daphnetin is achieved via GLP-1R-mediated oxidative stress and inflammation. To further explore the mechanism of GLP-1R, we transfected GLP-1R small interfering RNA (si-GLP-1R) into methylglyoxal-treated PC12 cells, and detected GLP-1R expression and the levels of inflammation and oxidation stress-related factors. As shown in Figures 5C–E, compared with the control group, methylglyoxal significantly reduced GLP-1R level, increased inflammatory factor (IL-1β, TNFα, IL-6, iNOS and COX2) levels, increased ROS content, and reduced antioxidant enzyme activities (SOD, CAT, GSH, GPx), while daphnetin-treated showed significant anti-inflammatory and antioxidant effects and increased GLP-1R expression. Furthermore, the anti-inflammatory and antioxidant effects of daphnetin were attenuated when GLP-1R was silenced. These results indicated that silencing of GLP-1R attenuates the neuroprotective effect of daphnetin.

### 3.6 Overexpression of GLP-1R enhanced the protective effect of daphnetin

**FIGURE 6:**
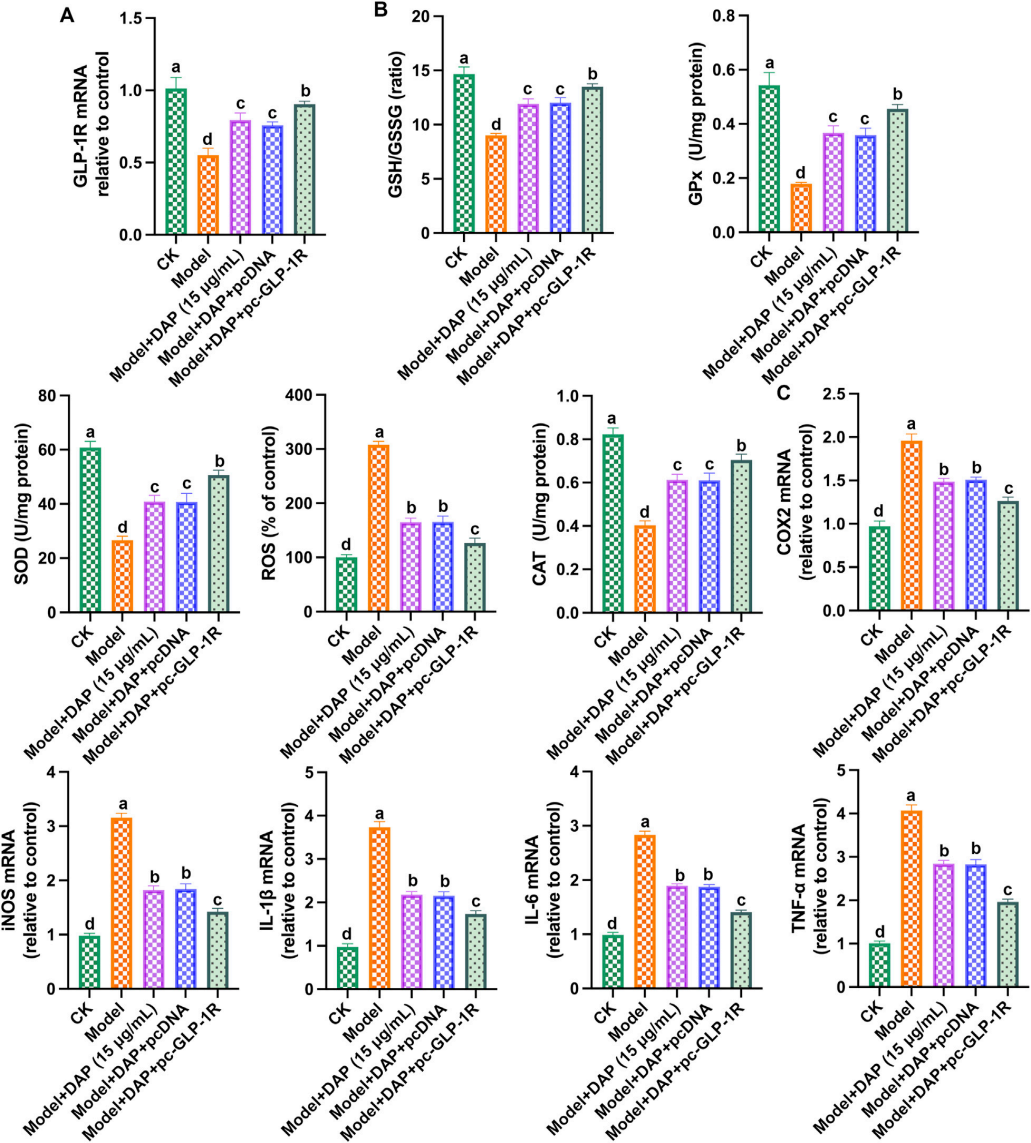
Overexpression of GLP-1R enhanced the protective effect of daphnetin. **(A)** GLP-1R mRNA expression; **(B)** mRNA expression of GLP-1R; **(C)** Levels of SOD activity, CAT activity, ROS content, GPx content and GSH/GSSG ratio; **(D)** mRNA expressions of COX2, TNFα, IL-6, IL-1β and iNOS; **(E)** mRNA expressions of COX2, TNF-α, IL-6, IL-1β and iNOS. Different letters **(A–D)** denote a significant difference between groups (n = 6) (*p* < 0.05).

Furthermore, we transfected GLP-1R overexpression plasmid (pc-GLP-1R) into methylglyoxal-treated PC12 cells. As shown in Figure 6, overexpression of GLP-1R enhanced the protective effect of daphnetin, reduced the levels of inflammatory factors, increased the levels of antioxidant enzymes, and upregulated the expression of GLP-1R. These findings strongly suggest that daphnetin alleviates diabetic cognitive dysfunction by modulating GLP-1R-mediated oxidative stress and inflammation.

## 4 Discussion

Diabetes, as a metabolic disease, has become a major public health problem. Diabetic cognitive dysfunction is one of the complications of diabetes and is affected by multiple factors, such as nervous system disorders, neuroinflammation and oxidative stress (Xiang et al., 2022). In recent years, the inflammation and oxidative stress theories have been widely recognized in the study of cognitive dysfunction (Choi et al., 2024; Mac Giollabhui, 2021). In recent years, the inflammation and oxidative stress theories have been widely recognized in the study of cognitive dysfunction. Previous studies have reported that increased oxidative stress in the brain of diabetic rats can lead to cognitive and behavioral defects (Ling et al., 2018), and increased ROS in the brain caused the dysfunction of antioxidant enzymes and weakened cognitive ability (Kumar and Menon, 1993). In the present study, we found that STZ and methylglyoxal treated increased the levels of inflammatory factor, promoted ROS generation, and reduced the activities of antioxidant enzyme in rats and PC12 cells. Additionally, we found that STZ-treated presented poor behavioral experimental results and increased blood glucose. These results demonstrated that diabetes-induced cognitive dysfunction and accompanied by inflammation and oxidative stress.

Glucagon-like peptide-1 (GLP-1) is an incretin hormone, glucagon-like peptide-1 receptor (GLP-1R) agonists play an important role in the treatment of diabetes (Gallwitz and Giorgino, 2021). GLP-1R agonists become research hotspot in diabetes-related medicine field and are used in the development of targeted drugs for diabetes. In the present study, we focused on the effect of GLP-1R on oxidative stress and inflammation in diabetes cognitive dysfunction. In PC12 cells and rats models, we indicated that the expression of GLP-1R is downregulated in diabetes, which demonstrated that GLP-1R is a critical factor in diabetes cognitive dysfunction.

Daphnetin is an active ingredient with broad application prospects and has been used in the treatment of various diseases (Syed et al., 2022). It is worth noting that Daphnetin has a good effect on diabetes and neurological diseases. Gao et al. (2022) stated that daphnetin could relieved Alzheimer’s disease-caused cognitive impairment through STAT3/GFAP pathway (Gao et al., 2022). Daphnetin were used to treat diabetic nephropathy (Ghosh et al., 2022) and STZ-caused β-cells damage (Vinayagam and Xu, 2017). In this study, we explored the pharmacological effects of daphnetin in diabetic cognitive dysfunction. We found that daphnetin has a significant anti-diabetic effect, which significantly reduces blood glucose in STZ-treated rats, and alleviates diabetic cognitive impairment, which proved by the improved behavioral test results. Furthermore, *in vitro*, daphnetin protected against methylglyoxal-induced PC12 cell damage, which proved that daphnetin exerted a neuroprotective effect.

Generally speaking, the mechanisms by which active ingredients such as daphnetin exert their health benefit are relatively complex, but are closely related to their antioxidant and anti-inflammatory properties. For example, daphnetin prevented chronic obstructive pulmonary disease through mitigating NLRP3-mediated inflammation and pyroptosis (Fan et al., 2023). Daphnetin alleviated cardiac remodeling in mice by exerting its antioxidant activity, with Nrf2/HO-1 and TGF-β1/Smad2/3 as key mechanisms (Syed et al., 2022). In the current study, we focused on the relationship between daphnetin’s anti-diabetic cognitive dysfunction and its anti-inflammatory and antioxidant properties, and explored the key regulatory role of GLP-1R in it. We found that daphnetin reduced blood glucose, mitigated diabetes cognitive dysfunction, and inhibited diabetes-caused inflammation and oxidative stress in brain. Further studies found that overexpression of GLP-1R enhanced the anti-diabetic effect of daphnetin and significantly reduced inflammation and oxidative stress, while silencing GLP-1R weakened the health benefits of daphnetin.

## 5 Conclusion

**FIGURE 7:**
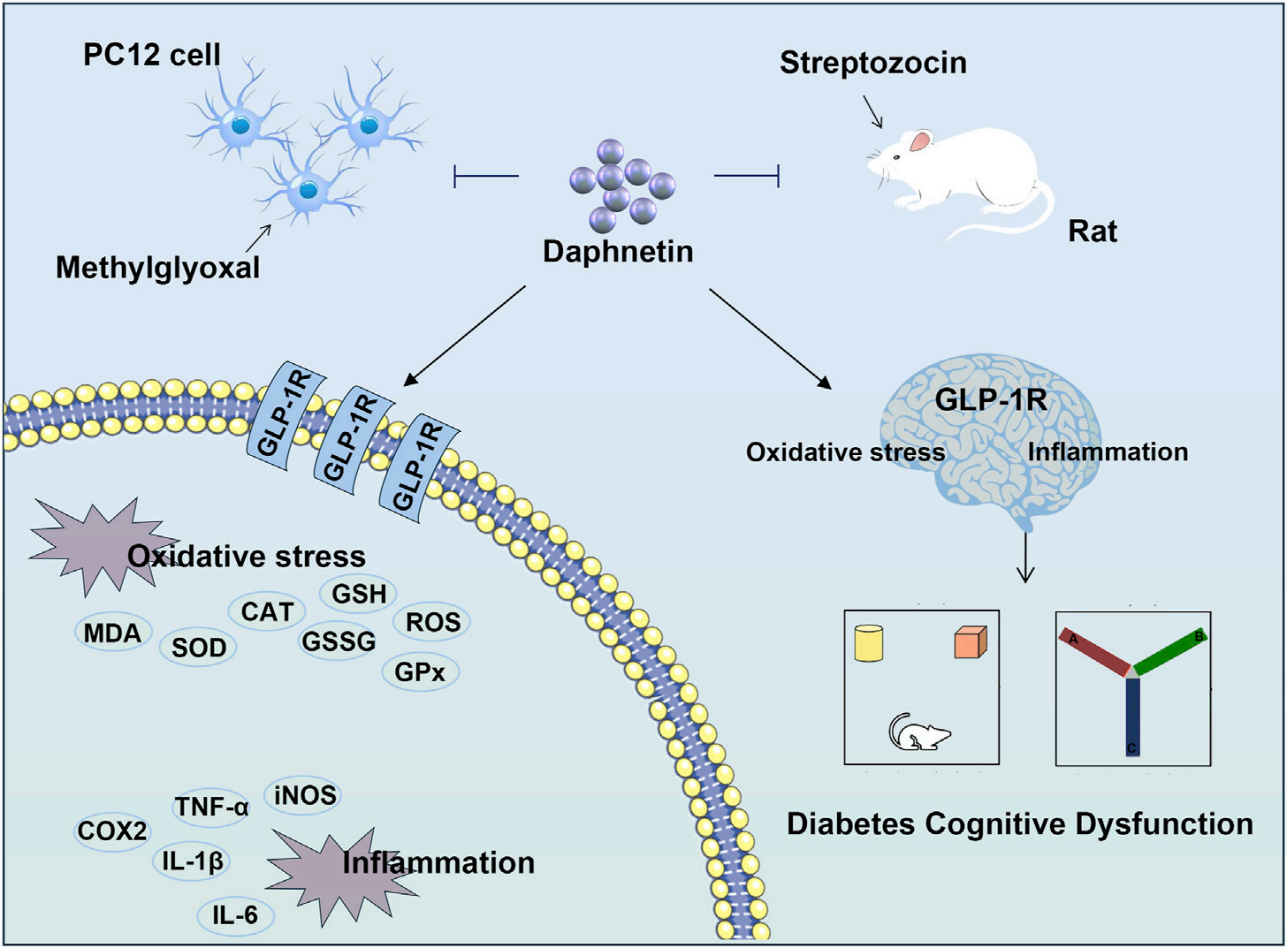
Daphnetin mitigates diabetes cognitive dysfunction through regulating GLP-1R-mediated oxidative stress and inflammation.

In a nutshell, daphnetin exerts anti-inflammation and anti-oxidation properties in diabetes cognitive dysfunction. Mechanically, daphnetin may exert its anti-diabetic cognitive dysfunction effect as a GLP-1R agonists (Figure 7). Our present study identifies an important role for GLP-1R in diabetes treatment, highlights the potential of natural plant-derived substances in the treatment of diabetic cognitive impairment, and provides new ideas for the future development of drugs to treat diabetic cognitive dysfunction.

## Data Availability

The original contributions presented in the study are included in the article/supplementary material, further inquiries can be directed to the corresponding author.
